# International eHealth ecosystems and the quest for the winning value proposition: findings from a survey study

**DOI:** 10.12688/openreseurope.14655.1

**Published:** 2022-05-06

**Authors:** Kira Oberschmidt, Lex van Velsen, Christiane Grünloh, Laura Fiorini, Erika Rovini, Francisco José Melero Muñoz

**Affiliations:** 1 Biomedical Signals and Systems Group, University of Twente, Enschede, 7500AE, The Netherlands; 2 eHealth department, Roessingh Research and Development, Enschede, 7500 AH, The Netherlands; 3 Department of Industrial Engineering, University of Florence, Florence, 50139, Italy; 4 Telecommunication Networks Engineering Group, Technical University of Cartagena, Cartagena, 30202, Spain; 5 Technical Research Centre of Furniture and Wood of the Region of Murcia (CETEM), Yecla, 30510, Spain

**Keywords:** eHealth ecosystem, cross-country, interoperability

## Abstract

Background: eHealth ecosystems are becoming increasingly important for national and international healthcare. In such ecosystems, different actors are connected and work together to create mutual value. However, it is important to be aware of the goals that each actor pursues within the ecosystem.

Method: This study describes the outcomes of a workshop (30 participants) and two surveys (completed by 54 and 100 participants), which investigated how different types of industry stakeholders, namely social services, healthcare, technology developers and researchers, rated potential value propositions for an eHealth ecosystem. Both the feasibility and the importance of each proposition was taken into account.

Results: Interoperability between services was highly valued across industry types but there were also vast differences concerning other propositions.

Conclusion: Jointly reflecting on the different perceived values of an ehealth ecosystem can help actors working together to form an ecosystem.

## Plain language summary

Technology use in healthcare contexts has increased in the last years, both within specific countries and on a more global level. Connecting different healthcare technologies to so called ‘ecosystems’ can have benefits for the users of such systems. But the benefits can differ for the various people that interact with such a system, like citizens, healthcare professionals or researchers. This paper describes the results from a survey regarding the benefits that different people see for technical ecosystems in healthcare. Participants from different fields, specifically social services, healthcare, technology developers and researchers, rated how important and feasible they thought different ideas for such ecosystems are. Their opinions varied, but all participants saw the benefits of having different technical services interact with each other in the ecosystem. 

## Introduction

In the last few years, we have witnessed the rise of eHealth ecosystems, both on a national and international level. An example of such an ecosystem is a service whereby patients with chronic obstructive pulmonary disease (COPD) are treated by the hospital, the General Practitioner, and the physiotherapist (each with their own health information systems), as well as a set of Internet of Things (IoT) technologies and wearables, all linked to each other and exchanging data. These ecosystems can improve the quality of healthcare in an age, especially for those users of health care systems from remote and rural areas with numerous connectivity issues ( Dimitrievski *et al.*, 2021), where the patient is at the center of attention and is surrounded by multiple (informal) care providers and where care is provided both on-site, as well as in the community, via traditional means and via technological innovations. An eHealth ecosystem can therefore be defined as a configuration of people, eHealth technologies, and other resources that interact with other service systems to create mutual value (definition based on ( Maglio *et al.*, 2009)). As such, an ecosystem has the goal to connect dispersed actors, organizations and technologies to create value that cannot be obtained when working in silos ( Vargo *et al.*, 2017). In an eHealth ecosystem, technology is used to connect actors on the micro level (e.g., medical specialists, patients, ICT managers), the meso level (e.g., hospitals, technology start-ups), and the macro level (e.g., government bodies, the WHO) ( Chandler & Vargo, 2011).

The benefits of a service ecosystem can be found in different directions. By linking different services (i.e., resource integration), new services can be offered, and end-users can access the different services more easily. Additionally, ecosystems improve the scalability of single services ( Sklyar *et al.*, 2019). Within the context of healthcare and eHealth, service ecosystems have been found to improve individual and public wellbeing by reconfiguring the interaction among the different players involved and thereby enabling value co-creation ( Sebastiani & Anzivino, 2021b). An inventory by Al-Shaqi and colleagues (2016) uncovered that most Active and Assisted Living (AAL) ecosystems have the goal to either improve daily activities and social connectedness, enhance older adults’ safety, or monitor their health. eHealth ecosystem services are most often used in the home setting, followed by use in medical centers ( Sadoughi *et al.*, 2020).

In order to reap the benefits of eHealth ecosystems, different authors have inventoried a set of challenges that need to be overcome. These challenges can have a technical nature, like managing complex and huge amounts of data, ensuring scalability, facilitating interoperability, optimizing security & privacy, and designing for high usability ( Asthana *et al.*, 2019; Farahani *et al.*, 2018; Nijeweme-d’Hollosy *et al.*, 2015). On the other hand, barriers are also present on the organizational and human level ( Fiorini *et al.*, 2022; Sebastiani & Anzivino, 2021a). Sebastiani and Anzivino (2021a) studied key inhibitors and enablers on these levels for an Italian eHealth ecosystem that supports care for patients with chronic cardiovascular problems during the COVID-19 pandemic. The main inhibitors they found were inter- and intra-actor misalignments (a lack of mutual understanding and the lack of a common worldview among the main actors involved), resource myopia (a lack of understanding the full potential of the tools that are implemented in an ecosystem), and the platformisation gap (the lack of a virtual platform through which actors can interact). The main enablers they identified were actor role empowerment (strengthening key players, like care professionals and patients, in the role they play in service provision), actor-network engagement (building new, meaningful interactions among actors on the micro, meso and macro level), and resource reconfiguration (the implementation of new and different ways to provide care).

The importance of a common worldview and a common goal, and the impact on the success of eHealth ecosystems when lacking these visions, make it a crucial first step to identify the goal(s) that the developers of the ecosystem should work towards ( Swinkels *et al.*, 2018). Breeman and colleagues (2021) conducted a series of qualitative studies to identify the values that an eHealth ecosystem for patients with cardiovascular conditions should fulfil. The values they elicited included autonomy support and personalization of the service offering. However, the value of an eHealth ecosystem is very context specific. Value will mean something different for actors on the micro, meso and macro level. Nonetheless, creating a shared worldview with a common set of goals that the ecosystem should serve is paramount for its success ( Frow *et al.*, 2019).

In this article, we report on a study with the aim to elicit value propositions for an international eHealth ecosystem for AAL among older adults, involving healhprofessionals and caregivers. More precisely, we inventoried potential value propositions in a workshop and a first online survey, and we ranked the importance of the potential value propositions among the ecosystem’s potential stakeholders. The results of this work enrich the small body of literature that is available on eHealth ecosystem development and guide the discussion on the goal(s) this ecosystem should serve. The remainder of this article is as follows. In the next chapter, we will introduce the study context (the Pharaon project) and our study methods. Chapter 3 contains our results, which we will discuss and link to the current literature in chapter 4. We end this article with our conclusions in chapter 5.

## Method

### Ethics

Ethical approval was not required for the study. Written informed consent for publication of the participants’ answers was obtained from the participants.

### Study context

**Figure 1.**
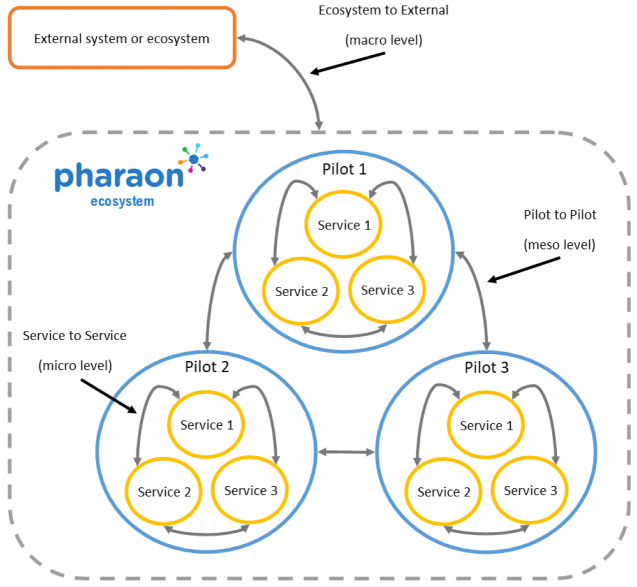
Schematic representation of the Pharaon ecosystem.

The Pilots for Healthy and Active Ageing ( Pharaon) project is a European project, involving 40 partners from 12 European countries, that aims to develop, implement and test an ecosystem to enable active and assisted living for Europe’s ageing population. It does so by creating a set of interoperable, customizable open platforms with advanced eHealth services and tools, such as medical devices, IoT, artificial intelligence, big data and analytics, and online services. In some sites, these technical services are supplemented by traditional, offline services. The open platforms will be implemented in eight pilot sites, situated in Spain (Andalusia and Murcia), Italy (Tuscany and Apulia), Portugal (Coimbra and Amadora), the Netherlands, and Slovenia. Figure 1 depicts a schematic representation of the Pharaon ecosystem.

Within each pilot, a set of AAL services are integrated (both on a data and functional level), supported by a local integration platform. Within the Pharaon ecosystem, pilots are also integrated to support data and service exchange. Finally, external ecosystems or systems can make use of the aggregated data collected in the Pharaon ecosystem to enrich their services.

### Study design

To determine the value propositions that the Pharaon ecosystem should serve, and hence to specify the common goal the partners within the ecosystem should strive for, we conducted a study in three parts:

**1.** **Internal workshop**. To shape the initial list of potential value propositions, we conducted an online workshop with a selection of the Pharaon project partners.

**2**. **Internal survey**. Then, we conducted an online survey among all people involved in the Pharaon project to assess which propositions they found most important and feasible, and to identify any missing value propositions.

**3. External survey**. Finally, we conducted an external survey among potential stakeholders of the Pharaon ecosystem to identify the most important and most feasible value propositions per stakeholder type.

***Internal workshop.*** The aim of this workshop was to pilot an initial list of value proposition statements and the methodology that was planned for the subsequent surveys. As a preparation for the workshop, an initial list of value propositions was created, based on internal work within the project, as well as literature ( Marguglio, 2020; Schiza *et al.*, 2019). The statements were presented sorted by the stakeholder that was expected to benefit from this value proposition, which was either Service and Technology providers, Older Adults or the European Union. There were ten value propositions, divided by stakeholder type as follows:

**Value for Service and Technology providers**

The Pharaon ecosystem can help service and technology providers to:

  ●   prove the quality of their product by providing a quality seal.
  ●   facilitate cross-country implementation and integration by making the connection to international partners and platforms.
  ●   increase the financial viability of their product by lowering the Total Cost of Ownership (TCO).
  ●   scale up their product by offering an international market place and a common Application Programming Interface (API).
  ●   increase the attractiveness of their product by facilitating connections with other technologies.

**Value for Older Adults**

The Pharaon ecosystem can help older adults to:

  ●   access a variety of connected technologies easily by integrating and connecting them.
  ●   access new technologies quickly by easily integrating new products into the ecosystem.
  ●   share information across systems and borders by linking all technologies within the system and by allowing data exchange between them.

**Value for the European Union**

The Pharaon ecosystem can help the European Union to:

  ●   gather large amounts of health data from various countries by facilitating data aggregation.
  ●   offer better healthcare for her citizens by facilitating cross-country use of working health technologies.

Thirty participants from the Pharaon project attended the workshop, which lasted for 45 minutes. Partners were invited based on their role in the pilot, focusing on those responsible for technical development and exploitation of the solutions, but pilots could also suggest other relevant participants. The workshop was held online, using the video conference tool Zoom and MURAL, a web-based tool for visual collaboration, to facilitate interaction among participants. The session started with a short introduction, after which the participants were grouped in six breakout rooms based on the pilot country they were involved in. They were presented with the set of value proposition statements in MURAL. For each statement, they were asked to rate the feasibility of creating it and the importance of the value proposition for their pilot site. Additionally, there was room to add any comments or remarks on the value proposition, and participants were encouraged to do so. There was also space to add general remarks and missing value propositions that came up in the discussion. At the end of the session, some key results were discussed in a plenary session.

***Internal survey.*** An internal survey was conducted within the project consortium between 3rd March and 12 ^th^ April 2021. Participants first answered demographic questions about their workplace, like the country and type of industry they work in and the number of employees in their organisation. Then they were presented with the ten value propositions that were rephrased and adapted from the internal workshop.

For each of the value propositions, the participants rated on a five point Likert scale (1) how feasible they thought this was, and (2) how important this was for their organisation. There was also room to add questions or remarks below each value proposition. In the last part of the survey, the participants were asked to select the value proposition that was the most valuable for them and their organisation. There was also room to add any value propositions they felt were missing.

 Descriptive statistics of the demographic variables were calculated. Participants’ answers on the importance and feasibility of each value proposition were split by type of industry. The mean score and standard deviation (SD) for each type of industry are reported. Answers to the question of which value proposition was the most valued overall were split per type of industry and visualized.

***External survey.*** The external survey started with the same organisational demographic questions as the internal survey. Again, participants were asked to rate the feasibility and importance of each value proposition for their organisation. In addition to the ten initial value propositions, the following two value propositions were added based on suggestions from the internal survey:

  ●   A pan-European ecosystem can support the collection of several types of citizen data (health and surroundings) in order to develop interventions for preventing premature ageing and improving quality of life.
  ●   A pan-European ecosystem can support pandemics (e.g., the COVID-19 crisis) by providing remote health and care services.

As in the first survey, there was room to leave a question or comment after each value proposition.

The survey was open between July and October 2021. Participants were recruited through the network of the organizations within the project, both via social media and personal invitation. The analysis of the survey was done in the same way as for the internal survey, reporting on demographics and visualizing the outcomes for the ratings of each value proposition per industry.

## Results

### Initial value proposition workshop

Based on the discussions and remarks during the workshop, it was decided to rephrase some of the value proposition statements slightly to make them easier to understand. Additionally, it was decided not to present the value propositions categorized by the benefitting stakeholder, because the value propositions often affected several actors at the same time. For example, the value proposition of ‘providing a seal of quality’ was categorized as beneficial for the Service and Technology providers, but participants pointed out that Older Adults could benefit from this as well. The final list of value propositions, which was then used for the internal and external survey, looked like this:

  ●   The Pharaon ecosystem can vouch for the quality of the services that it offers by providing a seal of quality.
  ●   The Pharaon ecosystem and its national platforms can facilitate cross-country implementation and integration.
  ●   The Pharaon ecosystem can increase the financial viability of included services by lowering the costs for integration and upscaling.
  ●   The Pharaon ecosystem can facilitate the upscaling of included services by offering an international market place and a set of common APIs.
  ●   The Pharaon ecosystem can increase the attractiveness of the associated services by facilitating interoperability with other services and/or technologies.
  ●   The Pharaon ecosystem can provide older adults with easy access to a variety of existing technologies by integrating and connecting them.
  ●   The Pharaon ecosystem can provide older adults with easy access to new, innovative technologies quickly by integrating new services into the ecosystem.
  ●   The Pharaon ecosystem can facilitate citizens to share personal health information across health systems and country borders.
  ●   The Pharaon ecosystem can support the gathering of large amounts of public health data from various countries.
  ●   The Pharaon ecosystem can support the provision of better healthcare for all European citizens by facilitating cross-country use of health services.

The participants considered the rating based on feasibility and importance as a good exercise to think about the value propositions in more detail, so it was decided to include similar questions in the surveys as well. Since the open comments also yielded many interesting insights, such room for comments was also included in the survey setup.

### Internal survey

***Demographics.*** Of the 86 participants that started the survey, 54 completed all questions. Only these 54 responses were included in the analyses. Participants from ten different European countries completed the survey. Most participants came from Portugal (n=12, 22.2%), followed by Spain (n=11, 20.4%) and Italy (n=10, 18.5%). The companies where participants were employed varied greatly in size, ranging from 1–4 employees (n=5, 9.3%) to more than 1000 (n=21, 38.9%). Most participants were employed by private companies, either non-profit (n=23, 42.6%) or for-profit (n=17, 31.5%). Participants most commonly worked in research and education (n=20, 37%), followed by social services (n=12, 22.2%) and technology development (n=10, 18.5%). In terms of the quadruple helix, the majority described their organization as industry / business (n=22, 40.7%). Academic research (n=15, 27.8%) and society (n=13, 24.1%) were also well represented, but only two participants from a state / government organization completed the survey (3.7%). Finally, the majority of participants described their role as research and development (n=30, 55.6%).

***Rating of importance.*** The ratings of the importance for each value proposition were sorted by type of industry of the participants’ organisation:

  1.   Social services (including public welfare, social work and universal healthcare).
  2.   Healthcare services (including primary, secondary and tertiary care).
  3.   Technology developers (including SMEs and businesses).
  4.   Researchers (including academics and educators).

Compared to the other industry types, participants from healthcare services provided the highest rating on all value propositions, sometimes more than a point higher than the ratings by other partners. Researchers also rated the importance of most value propositions much higher than the remaining industry types (social services and technology developers). The value proposition ‘easy access to a variety of technologies for older adults’ was among the highest scoring for three of the four industry types (technology developers: mean 4.10, SD 0.57; social services: mean 4.42, SD 1.00; healthcare services 4.67, SD 0.52). Another high scoring value proposition was ‘easy access to new, innovative technologies for older adults’ (researchers: mean 4.42, SD 0.90; social services: mean 4.00, SD 1.04). ‘Interoperability with other services and/or technologies’ received high ratings from technology developers (mean 4.20, SD 0.42) and social services (mean 4.00, SD 0.95) but was among the lowest rated by healthcare services (mean 4.33, SD 0.52). Similarly, researchers gave ‘Upscaling services by offering an international market place’ a high score (mean 4.45, SD 0.61), while the healthcare services’ rating was rather low (mean 4.33, SD 0.52). Conversely, healthcare services scored ‘sharing personal health data across health systems and country borders’ among the highest (mean 4.67, SD 0.52), while this value proposition was among the lowest scoring for the other three industry types (researchers: mean 3.40, SD 1.24; technology developers: mean 3.10, SD 0.57; social services: mean 2.75, SD 0.87). The ‘gathering of large amounts of Pan-European public health data’ received low scores from three of the four industry types (technology developers: mean 3.11, SD 0.78; social services: mean 3.25, SD 0.75; healthcare services: mean 4.00 SD 0.63).

***Rating of feasibility.*** Participants were asked to rate the feasibility of each value proposition from their organisation’s point of view. All industry types rated the value proposition ‘easy access to a variety of technologies for older adults’ the highest (researchers: mean 4.20, SD 0.95; technology developers: mean 4.10, SD 0.57; social services: mean 4.42, SD 0.67, healthcare services: mean 4,17, SD 0.75). The second highest ratings for researchers, technology developers and social services were all for the proposition of ‘interoperability with other services and/or technologies’ (researchers: mean 4.20, SD 0.70; technology developers: mean 4.00, SD 0.47; social services: mean 4.08, SD 0.52), whereas for healthcare services two other propositions scored highly: ‘cross-country implementation and integration’ and ‘sharing personal health data across health systems and country borders’ (both mean 4.17, SD 0.75). However, the value proposition of ‘sharing personal health data across health systems and country borders’ was among the lowest ratings from the other three industry types (researchers: mean 3.65, SD 0.93; technology developers: mean 2.90, SD 0.99; social services: mean 3.50, SD 0.80). Another low rated value proposition was ‘provision of better healthcare by facilitating cross-country use of health services’ (social services: mean 3.42, SD 1.00; healthcare services: mean 3.67, SD 1.03). Overall, technology developers rated feasibility the lowest in almost all cases.

**Figure 2.**
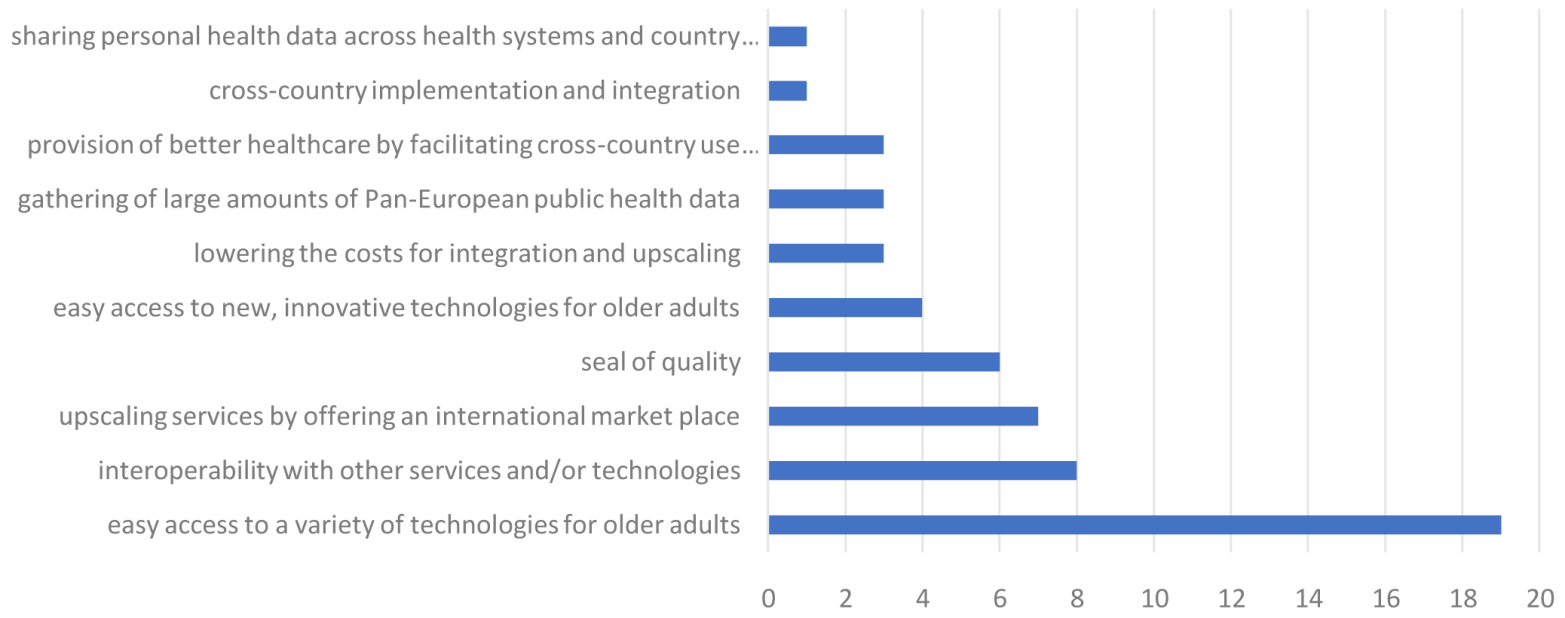
Rating of the most valued propositions within the Pharaon consortium.

Participants were also asked to rate the value proposition they valued most overall. These results are visualized in Figure 2. All value propositions were seen as the most valuable by at least one participant. However, the value proposition that received the most votes was that of providing ‘easy access to a variety of technologies for older adults’. When splitting the results by industry type, this value proposition was by far the most selected by social services (n=8, 66,67%) and also frequently selected by researchers (n=6, 30%).

Finally, there were two new suggestions for value propositions, one regarding the collection of citizen data to develop interventions and the other about remote health and care services during pandemics.

### External survey

***Demographics.*** 224 participants started filling in the survey, of which 100 completed all questions. Most participants came from Italy (n=38, 36.5%), Spain (n=15, 14.4%) and the Netherlands (n=10, 9.6%). Again, the size of the organization where participants were employed varied a lot, but a majority had more than 1000 employees (n=40, 35.5%). Most participants were employed at a private for-profit company (n=42, 37.2%). 41 participants (36%) work in research and education, followed by technology development (n=27, 23.7%) and health services (n=24, 21.1%).

***Ratings of importance.*** As for what the four types of organizations saw as important, there were several differences (see Table 1). Social services saw the value proposition of providing ‘remote health and care services’ as the most important (mean 4.56, SD 0.53), followed by ‘interoperability with other services and/or technologies’ (mean 4.33, SD 0.71). For the technology developers, the most important value propositions were ‘interoperability with other services and/or technologies’ (mean 4.33, SD 0.76) and ‘upscaling services by offering an international marketplace’ (mean 4.29, SD 0.75). Both the healthcare services and the researchers gave similar ratings for each proposition, without one value proposition clearly standing out. Healthcare service providers rated ‘interoperability with other services and/or technologies’ (mean 4.24, SD 0.63) highest, followed by ‘collection of citizen data (health and surroundings)’ and ‘remote health and care services’ (both mean 4.20, SD 0.70). For the researchers, ‘cross-country implementation and integration’ was the most important (mean 4.00, SD 0.87). ‘Remote health and care services’ received the second highest rating from these participants (mean 3.97, SD 1.10).

**Table 1.** Overview of the external participants’ ratings of importance per value proposition, split by industry type. The highest ratings per type of industry are highlighted in grey.

Value proposition		Importance rating (m, SD)			
		*Social* *services*	*Technology* *developers*	*Health* *services*	*Researchers*
1	Seal of quality	4.22 (0.83)	3.69 (1.01)	4.00 (0.76)	3.88 (0.85)
2	Cross-country implementation and integration	3.89 (0.60)	4.12 (0.82)	3.95 (0.90)	4.00 (0.87)
3	Lowering the costs for integration and upscaling	3.78 (0.83)	3.83 (1.05)	3.90 (0.63)	3.54 (1.17)
4	Upscaling services by offering an international market place	3.56 (0.73)	4.29 (0.75)	4.05 (0.90)	3.92 (0.94)
5	Interoperability with other services and/or technologies	4.33 (0.71)	4.33 (0.76)	4.24 (0.63)	3.90 (0.85)
6	Easy access to a variety of technologies for older adults	4.22 (0.67)	3.68 (1.09)	4.05 (0.89)	3.92 (1.01)
7	Easy access to new, innovative technologies for older adults	4.22 (0.67)	3.82 (1.14)	4.05 (0.87)	3.92 (0.97)
8	Sharing personal health data across health systems and country borders	3.78 (0.67)	3.57 (1.25)	3.86 (0.66)	3.68 (1.25)
9	Gathering of large amounts of Pan-European public health data	3.89 (0.78)	3.86 (1.39)	4.10 (0.72)	3.74 (1.20)
10	Provision of better healthcare by facilitating cross-country use of health services	4.11 (0.93)	3.82 (1.18)	3.95 (1.10)	3.79 (1.12)
11	Collection of citizen data (health and surroundings)	4.11 (0.93)	3.55 (1.22)	4.20 (0.70)	3.95 (0.96)
12	Remote health and care services	4.56 (0.53)	3.52 (1.29)	4.20 (0.70)	3.97 (1.10)

***Ratings of feasibility.*** The ratings on feasibility that the external participants provided were sorted by industry type (see Table 2). For most value propositions, the highest rating of feasibility was given by the social services industry. However, the value proposition ‘upscaling services by offering an international market place’ was rated lowest by the social services (mean 3.60, SD 0.70). Overall, the lowest ratings for feasibility came from the healthcare services. The ratings provided by the technology developers for each proposition were very close to each other, compared to the other groups that favoured particular propositions. The two most highly rated value propositions for social service participants were ‘remote health and care services’ (mean 4.60, SD 0.52) and ‘collection of citizen data (health and surroundings)’ (mean 4.33, SD 0.87). Technology developers and health services gave the highest ratings to the same two value propositions, namely ‘upscaling services by offering an international market place’ (technology developers: mean 4.12, SD 0.83; health services: mean 4.04, SD 0.88) and ‘interoperability with other services and/or technologies’ (technology developers: mean 4.12, SD 0.67; health services: mean 4.04, SD 1.11). Last, the researcher also highly rated ‘upscaling services by offering an international market place’ (mean 4.00, SD 0.81), followed by ‘gathering of large amounts of Pan-European public health data’ (mean 3.84, SD 1.08) and ‘cross-country implementation and integration’ (mean 3.80, SD 0.95).

**Table 2.** Overview of the external participants’ ratings of feasibility per value proposition, split by industry type. The highest ratings per type of industry are highlighted in grey.

Value proposition		Feasibility rating (m, SD)			
		*Social* *services*	*Technology* *developers*	*Health* *services*	*Researchers*
1	Seal of quality	4.10 (0.74)	3.78 (0.97)	3.54 (1.10)	3.63 (0.89)
2	Cross-country implementation and integration	4.00 (0.47)	3.96 (0.90)	3.50 (1.10)	3.80 (0.95)
3	Lowering the costs for integration and upscaling	3.90 (0.88)	3.84 (0.94)	3.41 (1.22)	3.39 (0.97)
4	Upscaling services by offering an international market place	3.60 (0.70)	4.12 (0.83)	4.04 (0.88)	4.00 (0.81)
5	Interoperability with other services and/or technologies	4.00 (0.67)	4.12 (0.67)	4.04 (1.11)	3.72 (0.86)
6	Easy access to a variety of technologies for older adults	4.10 (0.57)	3.78 (1.13)	3.68 (1.09)	3.59 (0.97)
7	Easy access to new, innovative technologies for older adults	3.90 (0.57)	3.70 (1.02)	3.55 (1.10)	3.58 (1.11)
8	Sharing personal health data across health systems and country borders	4.10 (0.74)	3.77 (1.31)	3.36 (1.36)	3.46 (1.17)
9	Gathering of large amounts of Pan-European public health data	4.10 (0.74)	4.00 (1.31)	3.57 (1.43)	3.84 (1.08)
10	Provision of better healthcare by facilitating cross-country use of health services	4.10 (0.57)	3.96 (0.98)	3.38 (1.28)	3.61 (0.95)
11	Collection of citizen data (health and surroundings)	4.33 (0.87)	3.91 (1.00)	3.76 (1.09)	3.66 (0.99)
12	Remote health and care services	4.60 (0.52)	3.78 (1.09)	3.67 (1.24)	3.76 (1.05)

**Figure 3.**
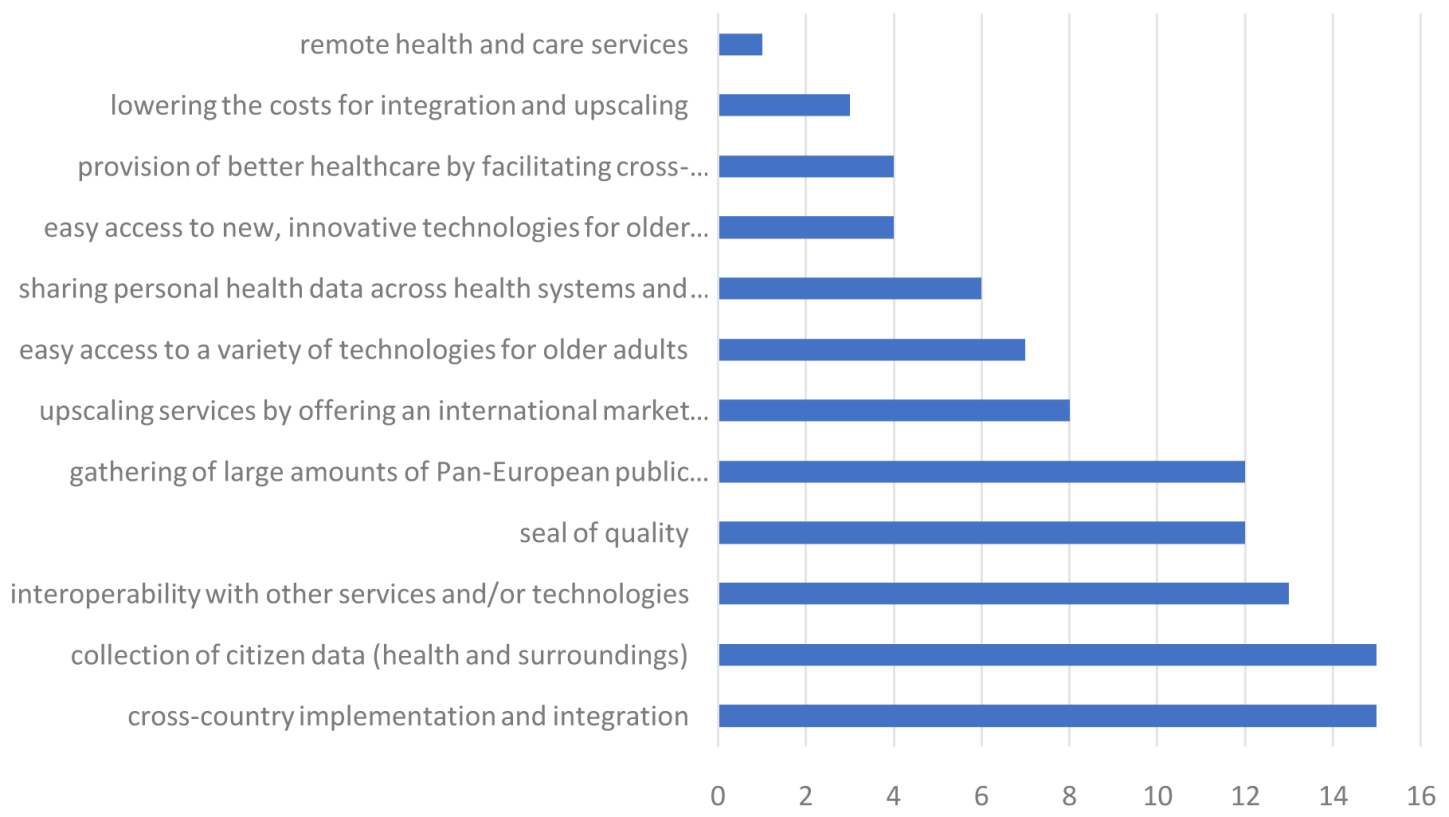
Rating of the most valued propositions by parties outside of the consortium.

***Most valued propositions.*** Again, each value proposition was scored as being the most important one by at least one participant. A visualisation of the scores for all value propositions can be found in Figure 3. The two most valued value propositions were ‘cross country implementation and integration’ and ‘collection of citizen data (health and surroundings)’.

The most valued propositions were split per type of industry partner to look for any differences as well. The researchers selected ‘cross country implementation and integration’ and the ‘gathering of large amounts of Pan-European public health data’ as most valued (both n=6, 17,65%). For all other industry types the answers were more divided.

**Figure 4.**
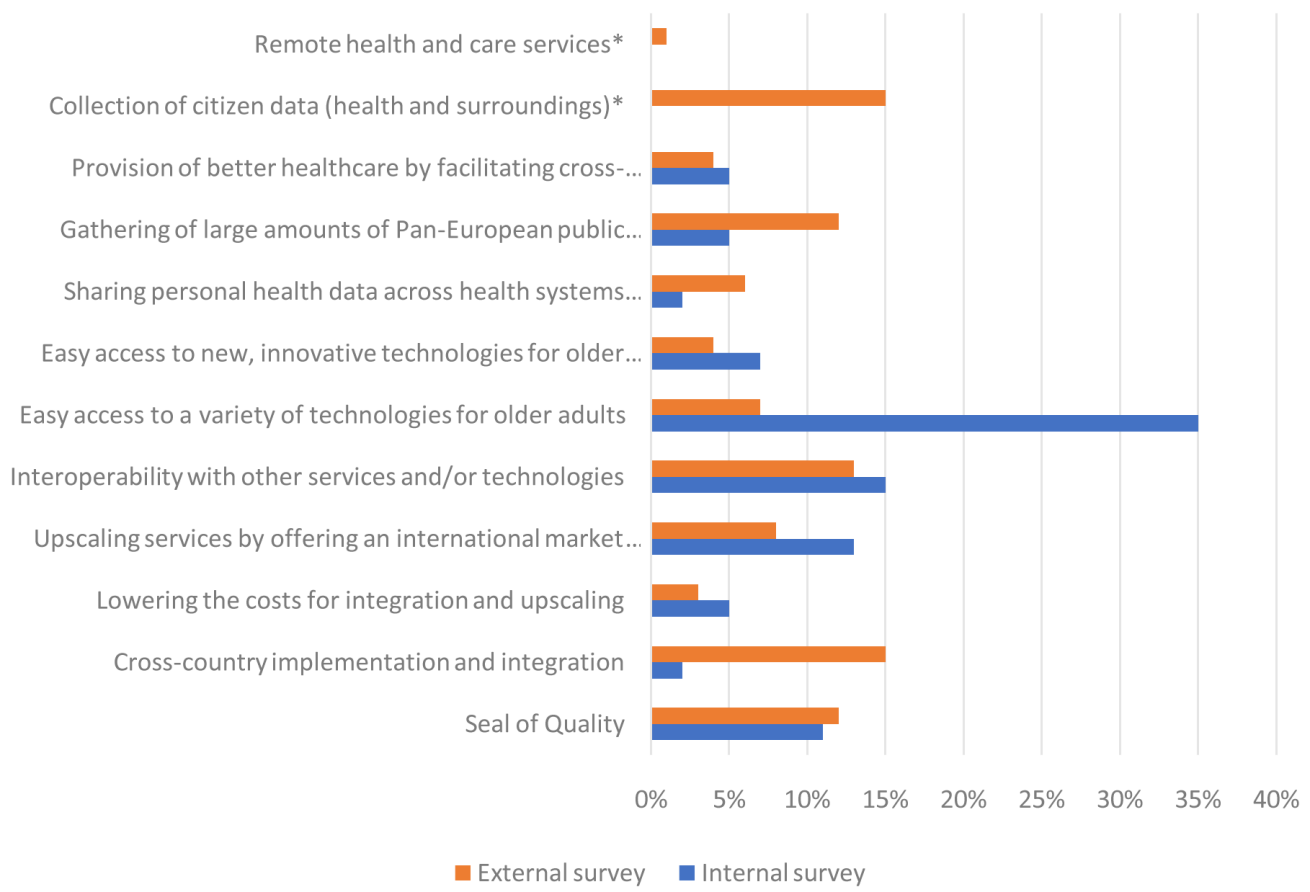
Percentage of the internal and external ratings of the most valued propositions. * *Value propositions marked with an asterisk were only presented in the external survey.*

Figure 4 shows the percentage of ratings that each value proposition received in the internal and external survey. The biggest difference can be seen for the rating of “easy access to a variety of technologies for older adults”, which was by far the most valued in the internal survey but was rated much lower in the external rating. On the other hand, ‘cross country implementation and integration’ and the ‘gathering of large amounts of Pan-European public health data’ received much higher ratings in the external compared to the internal survey.

## Discussion

This study aimed to elicit value propositions for a pan-European eHealth ecosystem and to investigate how relevant stakeholder groups rated the importance and feasibility of these value propositions. The value proposition that was rated as most important throughout the data collection was ‘interoperability with other services and/or technologies’. But in general, there was more division about most valued propositions.

As we discussed in the introduction, ecosystem literature often makes a distinction among the micro, meso and macro level, where different actors play a role. Previous work has also established that on the different levels, different types of value are desired ( Beirão *et al.*, 2017). For the context of this study, where we focus on an ecosystem to enable active and assisted living for Europe’s ageing population with the help of eHealth technology, this means that there is a difference between the gains that local care providers look for, the profit that technology developers seek, and the added value that (inter)national organizations desire. This specification of value was also found in our results. On the *micro level*, the main players are the health services, like hospitals and elderly care organizations, that provide services to older adults directly. The value they seek in an eHealth ecosystem is, on the one hand, to provide interoperable (eHealth) services of high quality that are easy to use, and on the other hand, to collect individual data (to monitor health). On the *meso level*, technology developers act as the glue among different sites that make up the ecosystem, and they should provide eHealth services and ensure functional and data integration. Their main aim is to increase their market by having more healthcare providers use their services (e.g., via an international marketplace as part of the ecosystem). On the *macro level*, social services act. They are mainly concerned with increasing public health and reducing healthcare costs through the use of high quality eHealth services.

The value proposition that was rated the highest throughout our surveys was ‘interoperability with other services and/or technologies’. This was seen as both important and feasible by the participants. However, other studies do not see interoperability as being feasible, but rather as a challenge of eHealth ecosystems ( Farahani *et al.*, 2018). This challenge stems from the fact that technology developers follow different regulations and standards that can be difficult to align with. Especially in the case of medical devices and technologies, where strict rules apply, this can make integration into an ecosystem laborious. A similar issue is emphasized by Lindquist and colleagues (2019) in their description of an ecosystem that connects applications to IoT technologies. In this case, different architectures make it difficult to connect services.

Another highly rated value proposition was the provision of ‘remote health and care services’, specifically in the light of the COVID-19 pandemic. This value proposition was suggested in the internal survey and related to the ability of using technology to provide healthcare at a distance, for example for patients in isolation. Blasioli and Hassini (2022) see similar possibilities for eHealth ecosystems, suggesting a connection between, for example, sensors and applications for monitoring health at a distance. Their description of an ecosystem therefore not only includes the value proposition of remote services but also those of providing easy access to new or existing technologies.

Ultimately, an eHealth ecosystem can only emerge and survive if it serves the values that are sought on the different levels by the different actors. It is therefore paramount that actors in an ecosystem acknowledge that different levels exist with different actors that pursue different goals, and give them the space to achieve these values. It remains a challenge, however, to streamline these actors and their intentions, as it has been found that actors, their characteristics, and their goals change over time, as actors grow alongside the ecosystem. Rather than trying to control these changes, it has been suggested to embrace them via an action research approach ( Vink *et al.*, 2021).

We also assessed the values that researchers thought were most important in an eHealth ecosystem. Since ecosystems are often the focus of research and innovation projects, being led by researchers, their interests might steer the design and development of the ecosystem, not without the risk of being in conflict with the needs of other stakeholders ( Oberschmidt *et al.*, 2020). As the results show, researchers do not seem to have an outspoken preference for a value proposition to be obtained, indicating that they are ambivalent towards the goal of the ecosystem itself and are therefore a suitable party to provide guidance in this endeavour. Interestingly the researchers’ estimates on the feasibility of obtaining the different value propositions were consistently among the lowest. This suggests that either researchers are too pessimistic on the realization of eHealth ecosystems, or that the other stakeholders in the ecosystem are too optimistic. We did observe differences in the value propositions that were considered as most desirable in an internal consulting round among partners of a research and innovation consortium that have committed to creating an eHealth ecosystem and similar partners that are not part of this initiative. This seems to suggest that there is a difference in values that are sought after by organizations that can be characterized as innovators and those that come afterwards (early adopters, early and late majority). It is therefore important to continuously map the value that the eHealth ecosystem should provide.

While there are examples of eHealth and healthcare ecosystems in literature, most of these focus on a regional or national instead of an international level ( Botti & Monda, 2020; García Holgado *et al.*, 2019; Schiza *et al.*, 2019). In these projects, many of the same value propositions are found as in this study, albeit on a smaller scale, connecting regionally instead of internationally. Examples include data gathering for geographical analysis ( García Holgado *et al.*, 2019), a national open data ecosystem ( Heijlen & Crompvoets, 2021), or the ability to access new technologies ( Botti & Monda, 2020). Schiza and colleagues (2019) describe a national level ecosystem that is aligned with EU standards (e.g., related to data protection and privacy), to allow for exchanging and connecting with other national ecosystems. This ecosystem mainly connects electronic patient records and facilitates exchange between healthcare professionals. The Pharaon project, however, aims to make a pan-European connection between various health technologies (new and/or existing) that users can access without the need to contact a healthcare professional. Therefore, while much can be learned from ecosystem projects on a smaller scale, knowledge on international ecosystems, which will become more and more important in an internationally connected society, is still scarce and should be extended in future research.

### Limitations

Like any study, this work has some limitations. First, the unbalanced division of participants over the different industry types, made it impossible to perform tests for statistical significance in the importance and feasibility scores that we obtained for the different value propositions. Second, the replies we gathered via the internal survey were most probably biased, as the participants were all members of a research and innovation consortium that focuses on developing an eHealth ecosystem. Therefore, we used their data as a pilot study for a larger and more objective inventory among organizations outside this consortium.

## Conclusion

Knowledge on developing international ecosystems in the healthcare domain is scarce. The added value of such an ecosystem is very context specific and depends on the various actors on the micro, meso and macro level. This study investigated the importance, feasibility and overall ranking of value propositions for a pan-European eHealth ecosystem, rated by relevant stakeholder groups from all levels. While the most important value proposition for the ecosystem was related to establishing interoperability with other services and/or technologies, the assessment of value propositions differed noticeably between actors on the micro, meso and macro level.

Considering that an ecosystem is a configuration of these actors and resources that come together to create value that cannot be achieved in silos, it is crucial to prevent misalignment and instead establish a common ground. This can be achieved by developing a joint value proposition that reflects the desired values of the actors from different levels. The joint reflection on the feasibility and importance of different propositions and goals was considered a useful exercise and can support actors to acknowledge that different actors exist on different levels who have a variety of goals. This establishes a common ground that subsequently can serve as the vision that all actors are working towards when developing an ecosystem that can be successfully implemented and operate sustainably.

## Data availability

### Underling data

DANS: EHEALTH ECOSYSTEM VALUE PROPOSITION SURVEY

https://doi.org/10.17026/dans-29m-25a7 ( Oberschmidt & van Velsen, 2021b)

This project contains the following underlying data:

● Pharaon+Ecosystem+Value+Proposition+-+External_October+20,+2021_08.09.ods
● Pharaon+Ecosystem+Value+Proposition_April+20,+2021_15.04.ods

### Extended data

DANS: EHEALTH ECOSYSTEM VALUE PROPOSITION IDENTIFICATION QUESTIONNAIRE

https://doi.org/10.17026/dans-z53-fnwj ( Oberschmidt & van Velsen, 2021a)

This project contains the following extended data:

eHealth_Ecosystem_Value Proposition_External Survey.pdf

eHealth_Ecosystem_Value Proposition_Internal Survey.pdf

Data are available under the terms of the Creative Commons Zero “No rights reserved” data waiver (CC0 1.0 Public domain dedication).

## Ethics and consent

Ethical approval was not required for the study. Written informed consent for publication of the participants’ answers was obtained from the participants.
