# Supplementary material for: International eHealth ecosystems and the quest for the winning value proposition: findings from a survey study
Source: Open Res Eur. 2022 May 6;2:56. [Version 1] doi: 10.12688/openreseurope.14655.1 (PMC10445862; doi:10.12688/openreseurope.14655.1)
Supplement: Peer review history

# Peer review: Reviewer response for version 1

**DOI:** 10.21956/openreseurope.15830.r31528

**Published:** 2023-05-16

**Authors:** Christine Jacob

**Affiliations:** 1 FHNW - University of Applied Sciences Northwestern Switzerland, Windisch, Switzerland

Thank you for the opportunity to review this relevant paper that aimed to elicit value propositions for an international eHealth ecosystem for Active and Assisted Living (AAL) among older adults, involving health professionals and caregivers. The manuscript is quite comprehensive and well structured, but there are few details that can be made clearer to the reader.

 Comments:

- The abstract doesn’t clearly inform the reader about the aims of the study, this should be added for clarity (i.e., to elicit value propositions for a pan-European eHealth ecosystem and to investigate how relevant stakeholder groups rated the importance and feasibility of these value propositions).
- The plain language summary only refers to the survey but not the workshop, it would be relevant to add it for accuracy.
- It’s not very clear in the manuscript how the workshop outcomes were analyzed and synthesized, this would be worth clarifying.
- Ethical considerations: it would be great if the authors could include a bit more details about how they ensured anonymity and privacy of the participants.

Is the study design appropriate and does the work have academic merit?

Yes

Is the work clearly and accurately presented and does it cite the current literature?

Yes

If applicable, is the statistical analysis and its interpretation appropriate?

Yes

Are all the source data underlying the results available to ensure full reproducibility?

Yes

Are the conclusions drawn adequately supported by the results?

Yes

Are sufficient details of methods and analysis provided to allow replication by others?

Yes

Reviewer Expertise:

eHealth adoption and implementation

I confirm that I have read this submission and believe that I have an appropriate level of expertise to confirm that it is of an acceptable scientific standard.

# Peer review: Reviewer response for version 1

**DOI:** 10.21956/openreseurope.15830.r31232

**Published:** 2023-05-10

**Authors:** Clarence Baxter

**Affiliations:** 1 Queensland University of Technology, Brisbane, Queensland, Australia

This article describes surveys regarding connecting healthcare technologies as ecosystems.

 The paper is well-written and clearly describes objectives, methodologies and outcomes arising from internal and external surveys conducted as part of the Pharaon project, in addition to an initial value proposition workshop.

 The study identified importance, feasibility and rankings of value propositions for a pan-European eHealth ecosystem reported by relevant stakeholder groups.

Minor:

- Ethics "Written informed consent for publication of the participants’ answers was obtained from the participants." - consider updating to "Written informed consent for publication of the participants’ de-identified answers was obtained from the participants."
- Internal Survey - Demographics - for the benefit of readers, please include reporting of survey response rate as percentage i.e. 62.8%.
- External Survey - Demographics - for the benefit of readers, please include reporting of survey response rate as percentage i.e. 44.6%.

Is the study design appropriate and does the work have academic merit?

Yes

Is the work clearly and accurately presented and does it cite the current literature?

Yes

If applicable, is the statistical analysis and its interpretation appropriate?

Yes

Are all the source data underlying the results available to ensure full reproducibility?

Yes

Are the conclusions drawn adequately supported by the results?

Yes

Are sufficient details of methods and analysis provided to allow replication by others?

Yes

Reviewer Expertise:

Telehealth, eHealth, mHealth, Public Health, Clinical Measurement Science

I confirm that I have read this submission and believe that I have an appropriate level of expertise to confirm that it is of an acceptable scientific standard.
